# Epidemiological and clinical profile of suicide attempts in Tunisian adolescents

**DOI:** 10.1192/j.eurpsy.2022.700

**Published:** 2022-09-01

**Authors:** R. Boukhchina, A. Aissa, I. Kammoun, Y. Zgueb, S. Madouri, U. Ouali, R. Jomli

**Affiliations:** 1 Razi hospital, Psychiatry A Department, Manouba, Tunisia; 2 Razi hospital, Psychiatry A Department, manouba, Tunisia

**Keywords:** suicide attempts, Suicide, adolescence, risk factors

## Abstract

**Introduction:**

Suicidal behavior remains an important clinical problem and a major cause of death in youth.

**Objectives:**

The purpose of this study was to describe the epidemiological and clinical profile of adolescents with suicide attempts.

**Methods:**

This is a retrospective descriptive study that focused on a population of Tunisian adolescents aged between 10 and 19 years old and who were hospitalized after a suicide attempt between between January, 1st 2010 and November,15th 2018, in Razi Hospital.

We used a pre-established questionnaire that explored the sociodemographic and clinical data of patients.

**Results:**

Sixty adolescents were included in this study. The average age of the respondents was 14.3±2 years. The sex-ratio (m/f ) was 0, 36. The suicidal adolescent was a female (73%), single (98%), enrolled in school (66%) with school failure history (52%). Family history of suicide was reported in 8%. Fifty adolescents (83%) lived with their parents and the relationship with them was described as disturbed in 60% of them. A history of physical and sexual abuse was reported in 25% during first adolescence and 15% during second adolescence. The most frequent diagnoses were adjustment disorder with depressed mood (45%) and depression (28%). Drug ingestion was the most common mean of suicide (63%), in an impulsive way in 82% of cases.

**Conclusions:**

Development of repeated epidemiological surveys makes it possible to better understand the prevalence of suicide attempts in adolescents and to implement suicide prevention programs.

**Disclosure:**

No significant relationships.

